# Case Report: A rare case of acute small bowel obstruction from a paraduodenal Treitz hernia: navigating diagnostic and surgical challenges

**DOI:** 10.3389/fsurg.2026.1761764

**Published:** 2026-04-15

**Authors:** Khaled Bajaeifer, Ghadah Sulaiman Alsaleh, Mohammed Alawi Alsakkaf

**Affiliations:** 1 General Surgery Department, Neom Hospital, Tabuk, Saudi Arabia; 2 Global Center for Mass Gathering Medicine, Ministry of Health, Riyadh, Saudi Arabia; 3 Emergency Medicine Department, Neom Hospital, Tabuk, Saudi Arabia

**Keywords:** internal hernia, laparotomy, paraduodenal hernia, small bowel obstruction, Treitz hernia

## Abstract

**Background:**

Internal hernias are a rare but critical cause of small bowel obstruction, with paraduodenal hernias being the most common subtype. They pose a significant diagnostic challenge due to non-specific symptoms and can lead to catastrophic outcomes like bowel strangulation.

**Objectives:**

This report details a case of acute small bowel obstruction secondary to a paraduodenal Treitz hernia to highlight the diagnostic and therapeutic challenges and discuss key management decisions.

**Case presentation:**

A 53-year-old male, smoker, with no surgical history, presented with severe progressive abdominal pain, vomiting, and constipation. Examination revealed abdominal tenderness and rigidity. Laboratory findings showed leukocytosis with neutrophilia, a markedly elevated creatine kinase and C-reactive protein. CT scan confirmed a small bowel obstruction with a tight transition point. An initial laparoscopic exploration was converted to open laparotomy due to poor visualization, revealing a non-strangulated paraduodenal Treitz hernia, which was successfully reduced. The patient's postoperative course was uncomplicated, with a rapid return to a liquid diet by postoperative day one.

**Conclusion:**

This case underscores that internal hernias must be considered in patients with small bowel obstruction and no prior abdominal surgery. Timely CT imaging is crucial for diagnosis, and surgical flexibility, with a readiness to convert to open laparotomy, is essential for safe management and optimal outcomes.

## 1 Introduction

Acute small bowel obstruction is a predominant surgical emergency, most frequently caused by postoperative adhesions, incarcerated external hernias, and neoplasms (1). Within this clinical landscape, internal hernias constitute a rare and diagnostically challenging etiology (2). They are defined by the abnormal protrusion of an abdominal viscus through a congenital or acquired aperture within the peritoneal cavity, without an external sac (3, 4). Internal hernias cause 1% of intestinal obstructions; within these, paraduodenal hernias which are the most common subtype, constitute 53% of internal hernias and are more frequent in men than women, with an incidence of 3 to 1 (5, 6). Paraduodenal hernias originate from congenital mesenteric defects arising from anomalous intestinal rotation during embryogenesis, manifesting specifically through the fossa of Landzert (left paraduodenal) or the fossa of Waldeyer (right paraduodenal) near the ligament of Treitz (7, 8).

The risk factors for developing a paraduodenal hernia are not well understood because the condition is so rare. However, they are primarily thought to be congenital, or they may develop after surgery or trauma. These hernias often do not cause any symptoms for a person's entire life. The first sign of a problem can be a sudden and life-threatening bowel obstruction in adulthood, with no prior warning (9).

If an internal hernia is not recognized or is managed incorrectly, the consequences can be severe. The condition can quickly escalate from a simple blockage to a closed-loop obstruction, where a segment of bowel is trapped at both ends. This can lead to strangulation, where the blood supply to the trapped bowel is cut off. Without blood flow, the bowel tissue becomes ischemic, leading to necrosis (tissue death). Once the bowel becomes necrotic, it can perforate, releasing intestinal contents into the abdomen and causing severe infection (sepsis), which is often fatal (2, 10, 11).

The objective of this comprehensive case report is to document the clinical journey of a patient with a paraduodenal Treitz hernia, providing a detailed narrative from initial presentation and diagnostic workup through to surgical intervention and postoperative management, with the aim of enhancing clinical recognition, discussing the complexities of radiographic interpretation, and reviewing the strategic considerations in the surgical management of this rare but serious condition to better equip clinicians in future encounters with similar challenging presentations.

## 2 Case presentation

### 2.1 Patient history & clinical findings

The patient was a 53-year-old male who presented to the emergency department with a chief complaint of severe abdominal pain for 1 day. The pain was described as stabbing in character, continuous, and progressive since its onset the previous evening. It was associated with vomiting, anorexia, and a history of constipation for 2 days. Critically, the patient reported no relieving factors for the pain, and the evaluating surgeon, notably documented that the patient's pain was “out of proportion” to the initial physical findings. The review of systems was negative for fever and negative for lower urinary tract symptoms. The patient's past medical history was unremarkable, with no known chronic illnesses, no history of recurrent abdominal pain, no regular medications, no known drug allergies, and no previous surgical history. His social history was significant for active smoking. A significant language barrier was noted, as the patient could not speak Arabic or English, which inherently complicated taking a detailed history and may have delayed the initial clinical evaluation of the severity of his condition. On physical examination, the patient appeared ill but he was alert and conscious. The vital signs were initially stable: Heart Rate 66 bpm, Respiratory Rate 16 breaths/min, SpO₂ 97% on room air, Temperature 36.9 °C, and Random Blood Sugar 160 mg/dL, apart from transient hypertension: (blood Pressure 170/91 mmHg). The abdominal examination revealed notable generalized abdominal tenderness and rigidity. This clinical picture immediately elevated concerns for a range of pathologies including mesenteric ischemia, perforated viscus, or an obstructive process like an internal or incarcerated hernia.

### 2.2 Diagnostic assessment & management

**Figure 1:**
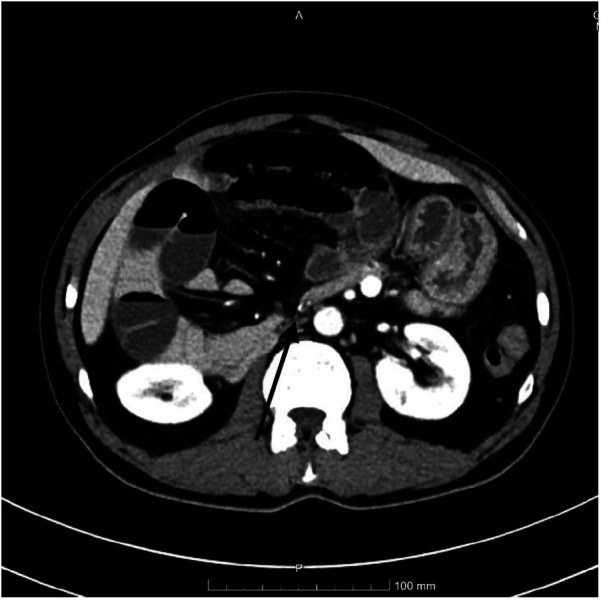
Contrast-enhanced abdomen/pelvis CT scan. A tightly narrowed transition point (2.7 cm) in the mid-abdomen, with proximally dilated small bowel loops and collapsed distal bowel, consistent with mechanical small bowel obstruction. The paraduodenal hernia defect is not directly visualized.

The diagnostic workup was initiated promptly upon the patient's arrival and was pivotal in guiding the subsequent urgent management. The cornerstone of diagnosis was a CT scan of the abdomen and pelvis with intravenous contrast, which was rapidly obtained. The radiological report described dilated loops of the small bowel, primarily the ileum, with a tightly narrowed transition point measuring approximately 2.7 cm in its long axis located in the mid-abdomen (Figure 1), features highly indicative of a mechanical small bowel obstruction. The absence of free fluid, pneumoperitoneum, or pneumatosis intestinalis was reassuring against frank perforation or advanced transmural necrosis at that moment. The CT also noted multiple mesenteric lymph nodes and emphysematous changes in the right lower basal lung region. Laboratory investigations revealed a leukocytosis of 12.73 × 10^9^/L, which further increased to 13.04 × 10^9^/L, with a profound neutrophilia (87.2%, absolute count 11.10 × 10^9^/L) and a relative lymphocytopenia (5.3%, absolute count 0.68 × 10^9/L), painting a clear picture of an acute inflammatory or stress response. This was supported by a significantly elevated C-reactive protein (CRP) level of 23.2 mg/L, further confirming the presence of a significant inflammatory state. Other notable laboratory abnormalities included a mildly elevated serum aspartate aminotransferase (AST) of 48.78 U/L and a markedly elevated creatine kinase (CK) of 925 U/L. Further laboratory data included a normal renal function (Creatinine 0.78 mg/dL, BUN 12.4 mg/dL), a slightly low serum sodium (135.7 mmol/L) and serum magnesium (1.51 mg/dL). Coagulation studies showed a mildly prolonged Prothrombin Time (16.0 s, INR 1.18) and a slightly low Partial Thromboplastin Time (30.2 s).

The patient was evaluated by the surgical consultant, and a decision was made for urgent laparoscopic exploration. The initial plan for a laparoscopic exploration was executed; however, upon entry into the peritoneal cavity, visualization was deemed poor and potentially hazardous, leading to the prudent decision to convert to an open laparotomy. The anesthesia was noted to be complicated by a difficult airway. The intraoperative findings confirmed the diagnosis of an internal hernia, specifically described as a non-strangulated Treitz hernia with overlapping intestinal loops herniating through a paraduodenal defect near the ligament of Treitz. Fortunately, the herniated bowel was viable and not strangulated, allowing for a successful reduction of the hernia contents back into the normal abdominal cavity. The patient received 3.5 L of crystalloid fluids intraoperatively with minimal blood loss and was transferred to the intensive care unit (ICU) for close postoperative monitoring.

### 2.3 Outcome & follow-up

The patient's postoperative course in the ICU and subsequently on the surgical ward was notably uncomplicated. On postoperative day zero, he was conscious, alert, and oriented (GCS 15/15). Examination revealed a laparotomy wound that was clean, not soaked, and soft/lax. His extremities were unremarkable. Sepsis markers indicated no signs of sepsis, and no antibiotics were given. His abdominal drain produced 200 mL of bloody output. His urine was concentrated, and he was on a fluid bolus. His laboratory parameters showed a trend improvement towards normalization. The immediate postoperative plan included NPO status except for sips of water, strict bed rest with head of bed at 45 degrees, DVT prophylaxis with Enoxaparin, adequate analgesia, maintenance of serum potassium above 4 mmol/L, routine labs, background IV fluids at 80 mL/hr, monitoring of hemodynamics and urine output, and monitoring of BP with consideration of antihypertensive medication if persistent elevation. By postoperative day one, he was successfully transferred out of the ICU, his nasogastric tube was removed as it was causing irritation and provoking cough, and he was started on a full liquid diet. He had not yet passed flatus or stool. His abdominal drain output had decreased significantly to a mere 10 mL of serosanguinous fluid, and his abdomen remained soft and non-tender. The plan for his care included continuation of prophylactic antibiotics, maintenance of drain in free flow and neutral position, strict input and output charting, incentive spirometry use, and ongoing DVT prophylaxis with Enoxaparin.

## 3 Discussion

The case of this 53-year-old male with an acute small bowel obstruction secondary to a paraduodenal Treitz hernia offers a wealth of educational points that underscore the diagnostic and therapeutic challenges inherent in managing rare abdominal pathologies. This case is challenging because internal hernias are difficult to diagnose. They often cause vague symptoms that mimic more common conditions, and diagnosing them requires a high index of suspicion, particularly in a patient like ours, with no history of abdominal surgery to suggest a more common cause like adhesions. Navigating this clinical scenario demands a synthesis of sharp clinical skills, careful interpretation of diagnostic clues, and decisive surgical judgment to avert a potentially catastrophic outcome.

One of the most profound unique points in this case was the presence of a significant language barrier, which added a layer of complexity to the initial diagnostic process. One of the most profound unique points in this case was the presence of a significant language barrier, which complicated the initial diagnostic process. Such a communication gap risks delayed diagnosis, misinterpreted symptoms, and challenges in obtaining informed consent, underscoring the critical need for professional interpreter services to guarantee patient safety and equitable care in diverse healthcare settings (12). This experience serves as a critical reminder that effective communication is not an administrative luxury but a fundamental component of patient safety, and institutions must prioritize access to professional interpretation services. A systematic review by Al Shamsi et al. (2020) confirms that language barriers systematically reduce satisfaction for both patients and providers and can decrease the quality and safety of healthcare delivery (13). The relevance of this issue in the local context is highlighted by a recent study from Makkah, where Bakhsh et al. (2024) found that a quarter of emergency department physicians always encounter language barriers, and over half attributed poor patient outcomes directly to communication difficulties (14). These findings validate the significant risk presented in our case and underscore that implementing solutions, such as the online translation tools suggested by Al Shamsi et al. (2020) (13), is an urgent operational necessity for modern emergency and surgical departments.

Furthermore, the laboratory profile upon presentation provided crucial, although non-specific, clues to the severity of the condition. The significant leukocytosis with profound neutrophilia and elevated C-reactive protein were clear markers of a significant inflammatory state, consistent with an acute abdominal process. More notably, the markedly elevated creatine kinase (CK) level, while potentially multifactorial, raised the suspicion of early muscle ischemia or substantial tissue injury, serving as a biochemical red flag that corroborated the clinical finding of “pain out of proportion” and heightened the urgency for intervention. The importance of this lies in reinforcing that laboratory studies, particularly a markedly elevated CK in the context of an acute abdomen, should not be dismissed and can serve as a critical objective data point that supports aggressive diagnostic and surgical management, even when other signs are still evolving. This is in contrast to the case reported by Shah et al. (2024), where laboratory findings were significant only for leukocytosis (15), and that by Alshdaifat et al. (2025), which also reported unremarkable labs aside from leukocytosis (16). The presence of a significantly elevated CK in our patient, while not pathognomonic, provided a more compelling objective marker of tissue-level distress, reinforcing the clinical suspicion of a compromised bowel and illustrating how a broader laboratory panel can refine risk stratification in ambiguous presentations.

Another critical and highly educational aspect was the radiographic findings on the CT scan. The identification of a “tightly narrowed transition point” in the mid-abdomen without an obvious lead-point mass or signs of inflammation is a classic, although rare, radiographic signature of an internal hernia. In a paraduodenal hernia, the bowel loops are often clustered in a sac-like formation in the paraduodenal area, and the mesenteric vessels can appear crowded or engorged; the radiologist's report in this case correctly identified the obstruction but the specific diagnosis of an internal hernia is often made in conjunction with the surgical findings, demonstrating the importance of a close dialogue between radiologists and surgeons. This underscores the necessity for surgeons to personally review imaging and engage in direct consultation with radiologists when clinical and radiographic findings are discordant or point to a rare etiology. The critical role of CT is echoed across the literature. In the case by Al Otaibi et al. (2019), CT clearly demonstrated a left paraduodenal hernia, facilitating a timely laparotomy (17). Furthermore, Lamprou et al. (2022) detailed specific CT signs of a right paraduodenal hernia, including an abnormal relationship between the superior mesenteric artery and vein and intestinal malrotation (18). Conversely, the case by Wassef et al. (2025) demonstrates the potential for diagnostic error, as the initial CT was deemed normal, and the hernia was only identified retrospectively after a second scan following recurrent symptoms (19). These cases collectively highlight that while CT is indispensable, its interpretation requires expertise and a high index of suspicion for internal hernias, as understated signs can be easily overlooked, leading to dangerous delays.

A pivotal point of discussion further revolves around the intraoperative decision-making. The initial choice for a laparoscopic approach was sound, aligning with modern surgical principles of minimally invasive surgery which offer benefits of reduced postoperative pain and faster recovery. However, the surgeons' willingness to convert to an open laparotomy upon encountering poor visualization was a crucial and commendable decision. In the context of a possible internal hernia with congested, dilated bowel, laparoscopic reduction can be hazardous, risking iatrogenic enterotomy or incomplete reduction, and the conversion to an open procedure allowed for safe manual reduction of the hernia and definitive assessment of bowel viability, which was paramount to the patient's successful outcome. This case therefore highlights that surgical judgment and the safety-first principle of converting to an open procedure should always surpass a rigid commitment to a minimally invasive approach, especially in complex and uncertain situations. This careful approach is supported in other case reports. Similar to our case, Lamprou et al. (2022) initially attempted a laparoscopic approach but converted to an open laparotomy upon encountering dilated loops and hemorrhagic fluid (18). In a case of a different internal hernia, Arif & Mohammed (2021) also converted from laparoscopy to open surgery due to difficulties visualizing organs amidst dilated bowel (20). These consistent experiences across different hernia types suggest that conversion is a common and often necessary step for safe management. In contrast, Wassef et al. (2025) successfully used a laparoscopic approach for a chronic case, but notably, their patient required a second operation for recurrence, potentially illustrating the technical challenges of definitive laparoscopic repair in the acute setting (19).

A key factor in the favorable outcome was the non-strangulated nature of the hernia at operation. The absence of bowel ischemia eliminated the need for resection, which significantly reduces postoperative morbidity, the risk of anastomotic leak, and the duration of recovery. This underscores the critical importance of timely intervention before the progression to strangulation. The subsequent uncomplicated postoperative course, characterized by rapid return of bowel function and the ability to initiate a liquid diet by postoperative day one, is a direct testament to achieving surgical correction before the onset of irreversible ischemic damage. The central lesson here is that the window of opportunity for a best-case outcome in small bowel obstruction is narrow, and prompt management is the single most important factor in preventing bowel loss and its associated outcomes. The devastating consequences of delay are illustrated by cases where patients presented with advanced ischemia. For example, Alsairafi (2022) reported a 51-year-old male with a left paraduodenal hernia who required resection of a 10 cm gangrenous bowel segment (21). Similarly, Xu et al. (2020) described a case with extensive intestinal ischemia necessitating bowel resection, reflected in markedly abnormal laboratory values including elevated lactate and profound leukocytosis (22). These cases stand in sharp contrast to the outcomes in our patient and in those reported by Shah et al. (2024) (15) and Al Otaibi et al. (2019) (17), where timely intervention on viable bowel led to uneventful recoveries. This spectrum of outcomes powerfully reinforces that delay is detrimental; the difference between a simple reduction and a life-threatening resection is determined by early recognition and intervention.

The final unique element was the patient's demographics and history, a middle-aged male, a smoker, but otherwise healthy. This profile defies the typical patient with a small bowel obstruction, who often has a history of prior abdominal surgery. This absence should always trigger a broader differential diagnosis that includes internal hernias, as well as other rarities like gallstone ileus or malignant obstruction. Additionally, the patient's status as a smoker is an important comorbidity. Smoking is a well-established risk factor for impaired wound healing and pulmonary complications (23). The documented emphysematous changes on CT scan and the need for incentive spirometry postoperatively highlight the added layer of risk and the necessity for proactive pulmonary care in such patients to prevent post-operative pneumonia, especially after a laparotomy. This reinforces that a patient's social history, particularly smoking, must actively inform perioperative risk stratification and management to alleviate preventable complications. The demographic profile of our patient, a middle-aged male, is consistent with several other reported cases, such as the 51-year-old male described by Alsairafi (2022) (21) and the 38-year-old male in Xu et al. (2020) (22). However, it is noteworthy that paraduodenal hernias frequently present in younger adults as well, as seen in the cases of the 21-year-old male (Wassef et al., 2025) (19), the 24-year-old female (Al Otaibi et al., 2019) (17), the 26-year-old male (Shah et al., 2024) (15), and the 31-year-old male (Alshdaifat et al., 2025) (16). The consistent theme across all these wide range ages, including our own, is the lack of prior abdominal surgery, which should be the primary trigger to expand the differential diagnosis beyond adhesions to include congenital and internal causes of obstruction.

### 3.1 Strengths and limitations

The primary strength of this case report lies in its detailed documentation of a rare clinical entity from presentation to resolution, providing a clear clinical pathway for others to follow. A further strength is the detailed discussion of the intraoperative decision-making process, particularly the critical choice to convert from laparoscopy to laparotomy. This provides a practical and potentially practice-informing example of managing complex situations where patient safety must override a commitment to a minimally invasive approach. A limitation, inherent in all case reports, is its nature as a single patient experience, which limits the generalizability of the findings. Additionally, the lack of long-term follow-up to confirm the durability of the repair remains a limitation, although the immediate postoperative course was exemplary.

## 4 Conclusion

This case highlights that internal hernias must be considered in patients with acute small bowel obstruction and no prior abdominal surgery. Timely CT imaging is crucial for diagnosis. Surgical management requires flexibility, with a readiness to convert from laparoscopy to open laparotomy to ensure patient safety and optimal outcomes.

## 5 Implications for clinical practice

Clinicians should maintain a high index of suspicion for internal hernias in cases of obstruction without surgical history. Close collaboration between surgeons and radiologists is essential for accurate CT interpretation. Surgical teams must prioritize patient safety, viewing conversion to open surgery as a prudent step rather than a failure when managing these complex cases.

## Data Availability

The raw data supporting the conclusions of this article will be made available by the authors, without undue reservation.
